# Minimally invasive laser Doppler flowmetry is suitable for serial bone perfusion measurements in mice

**DOI:** 10.1016/j.bonr.2019.100231

**Published:** 2019-11-22

**Authors:** Nicholas J. Hanne, Elizabeth D. Easter, Jacqueline H. Cole

**Affiliations:** a Joint Department of Biomedical Engineering, University of North Carolina, Chapel Hill, NC, USA, and North Carolina State University, Raleigh, NC, USA; b Materials Science and Engineering, North Carolina State University, Raleigh, NC, USA

**Keywords:** Laser Doppler flowmetry, Bone blood perfusion, Vascular supply, Minimally invasive, Serial measurement, In vivo, LDF, laser Doppler flowmetry

## Abstract

In vivo laser Doppler flowmetry (LDF) has previously been used to quantify blood perfusion accurately at a single timepoint in the murine tibial metaphysis. However, this procedure entailed substantial disruption to soft tissues overlying the bone and caused notable localized inflammation for several weeks after the procedure, impeding serial measurements in the same mouse. In this study, we tested a less invasive technique to measure perfusion in the tibia with LDF and determined that it can be used serially in the same mouse without causing signs of inflammation or gait perturbations. Twenty 14-week-old C57Bl/6J mice were evenly divided into groups that either had daily treadmill exercise or remained sedentary. Within these activity groups, mice were evenly subdivided into groups that received LDF measurements either weekly or only once at the study endpoint. Bone perfusion was measured with LDF in the anteromedial region of the right tibial metaphysis. Serum concentrations of interleukin 6, incision site wound area, and interlimb coordination during gait were measured weekly for four weeks. Tibial perfusion did not differ significantly between exercise and sedentary groups within the weekly or endpoint-only LDF groups at any timepoint. Perfusion was significantly increased in the third week in the weekly LDF group relative to measurements in the second and fourth weeks. Ligation of the femoral artery caused consistent, rapid reductions in tibial perfusion, validating that LDF is sensitive to changes in tibial blood supply. Weekly LDF procedures did not adversely affect gait, as interlimb coordination during treadmill locomotion was similar between weekly and endpoint-only LDF groups at every timepoint. Images of the incision site show wound closure within one week, and serum concentrations of interleukin 6 were not significantly different between weekly and endpoint-only groups. Together, these findings demonstrate that our minimally invasive LDF technique is suitable for serial in vivo measurements of intraosseous blood perfusion without inducing localized inflammation or negatively affecting gait patterns in mice.

## Highlights

• Our modified, minimally invasive laser Doppler flowmetry (LDF) technique enables measuring bone perfusion weekly in mice.
• Weekly LDF procedures did not induce inflammation or alter gait patterns, which could otherwise confound bone studies.
• Ligation of the femoral artery confirmed that the LDF technique measures functional perfusion within the bone.

## Graphical abstract

**Figure:**
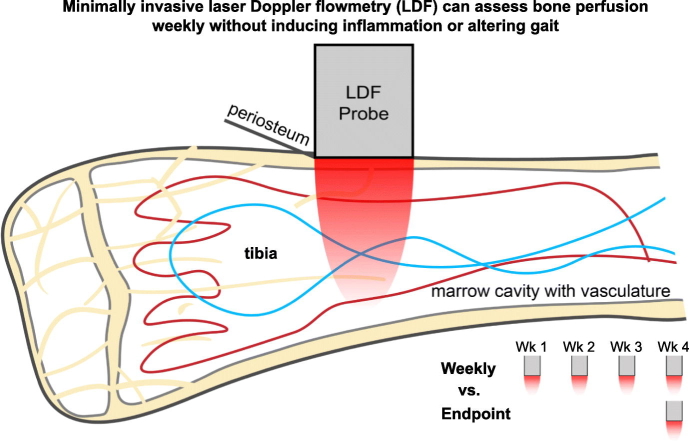

## 1 Introduction

Vasculature within bone (*osteovasculature*) is an essential contributor to bone health, providing nutrients, oxygen, cells, and chemical signals and removing waste products (Parfitt, 2000; Trueta, 1963). Adequate vascular perfusion is required for bone development, adaptation in response to loading, and healing after fracture (Trueta, 1963; Tomlinson et al., 2013; Wallace et al., 1991). Evidence that vascular pathologies are associated with bone loss is growing. Aortic calcification is associated with decreased lumbar spine bone mineral density (BMD) and increased fracture risk in men and women within four years (Naves et al., 2008), and the incidence of cardiovascular disease increases with reduced BMD in the spine in white men, and hip, trochanter, and femoral neck in black women (Farhat et al., 2007). Osteoporosis is associated with reduced perfusion in the vertebrae for men (Griffith et al., 2005) and in the femoral head for women (Griffith et al., 2008), although the mechanisms responsible for bone loss in these individuals is unknown and needs further examination using animal studies. Although murine models are commonly used to determine the effect of pathologies on bone properties, measuring blood perfusion within mouse bone is complicated due to their small size. Current methods are either experimentally difficult (e.g., hydrogen washout (Whiteside et al., 1977)) or require the animal to be sacrificed (e.g., microspheres, radiolabels, polyoxometalates, barium sulfate, or Microfil® (Serrat, 2009; Grundnes and Reikerås, 1992; Reeve et al., 1988; Kerckhofs et al., 2018; Barou et al., 2002; Yao et al., 2004; Boerckel et al., 2011)). Some methods can be performed in vivo but provide poor resolution not suitable for small bones: laser speckle imaging (Briers et al., 2013), laser Doppler perfusion imaging (Roche et al., 2012), contrast-enhanced magnetic resonance imaging (MRI) (Dyke and Aaron, 2009), contrast-enhanced positron emission tomography (PET) (Dyke and Aaron, 2009), and contrast-enhanced micro-computed tomography (Au et al., 2013; Clark et al., 2013). Endpoint and ex vivo measurements only provide a snapshot of vascular network function, missing the timing of vascular changes and any transient changes to vascular supply. A technique that could be used for longitudinal studies of bone perfusion in vivo would enable us to capture temporal changes in bone perfusion for individual subjects, thereby improving understanding of disease progression and intervention effectiveness.

First proposed as a tool to measure intraosseous perfusion by Nilsson et al. (1980), laser Doppler flowmetry (LDF) directs a monochromatic light source over a perfused tissue and measures backscattered light from fluid movement with a photodetector to provide a relative measure of blood perfusion. Perfusion is a functional measure of blood flow that is affected not only by the amount and velocity of red blood cells but also capillary density, vascular permeability, and flow direction (Swiontkowski, 1991; Griffith, 2014). LDF was first used to measure blood perfusion in the cancellous bone of pig mandibles by Hellem et al. (1983) and thereafter was rapidly adopted in orthopaedic clinics as an intraoperative tool to aid surgeons in identifying non-viable bone for debridement in patients with osteomyelitis, osteonecrosis of the femoral head, and lower limb traumatic injury (Swiontkowski, 1991). LDF has also been used as an endpoint measure to compare relative intraosseous perfusion between groups in murine research studies (Okunieff et al., 1998; Melnyk et al., 2008). Recently, it was used to quantify perfusion accurately in vivo in the mouse tibia, but the technique used in that study involved a relatively large incision that resulted in inflammation at the incision site up to three months after the procedure (Roche et al., 2013). To monitor longitudinal changes in murine bone perfusion, a less invasive LDF procedure is needed that will not induce significant localized inflammation or limping during gait, which could have both biological and mechanical confounding effects on bone.

We developed a minimally invasive LDF procedure and have used it to measure changes in tibial perfusion in mice in response to diet-induced obesity, ischemic stroke, and treadmill exercise (Hanne et al., 2019a; Hanne et al., 2019b). The objective of this study was to determine if this modified LDF procedure could be performed repeatedly in a longitudinal study without affecting bone perfusion, inducing inflammation, or altering limb coordination during locomotion, which could confound bone metrics of interest and perfusion changes associated with interventions. Developing new in vivo techniques to measure bone blood perfusion is a critical step needed to understand longitudinal changes to osteovasculature, which may contribute to bone loss occurring during progression of various clinical pathologies.

## 2 Materials and methods

### 2.1 Study design

**Fig. 1:**
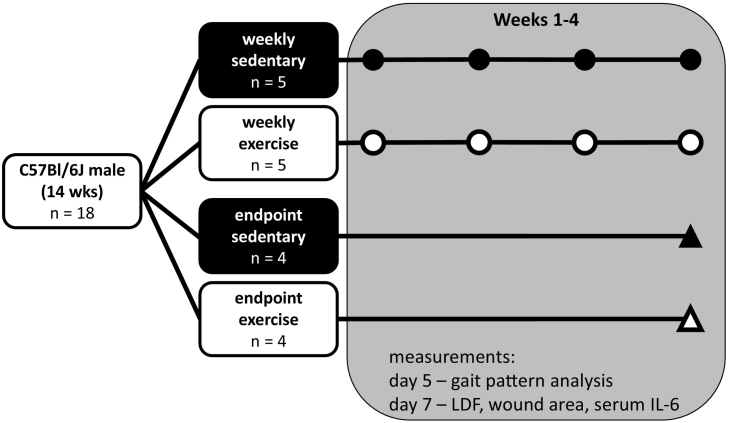
Experimental design. Symbols indicate weeks in which tibial perfusion was measured with LDF. Wound area, serum interleukin 6 (IL-6), and gait patterns were assessed weekly.

The protocol for this study was approved by the Institutional Animal Care and Use Committee at North Carolina State University. Eighteen 14-week-old male C57Bl/6J mice (The Jackson Laboratory, Bar Harbor, ME) were acclimated to the animal facility for one week. They were group-housed (4 per cage) on a 12-hour light/12-hour dark diurnal cycle and provided chow and water ad libitum. The mice were randomly assigned to four groups (Fig. 1) based on LDF procedure frequency (weekly, endpoint) and exercise regimen (sedentary, exercise): weekly sedentary (n = 5), weekly exercise (n = 5), endpoint sedentary (n = 4), and endpoint exercise (n = 4). Exercise groups were acclimated to treadmill exercise (Exer-3/6, Columbus Instruments, Columbus, OH) by increasing exercise intensity for 2 days prior to the start of the study (Day 1: 5 m/min for 10 min, 9 m/min for 10 min, and 12 m/min for 10 min; Day 2: 5 m/min for 5 min, 9 m/min for 5 min, and 12 m/min for 20 min). During the study, exercise groups performed daily exercise for 4 weeks (30 min/day, 5 days/week, 12 m/min, 5° incline), while sedentary groups were placed on a stationary treadmill for the same amount of time to equalize handling among the groups.

Laser Doppler flowmetry was used to measure intraosseous blood perfusion in the right tibial metaphysis, performed either weekly for 4 weeks (weekly groups) or at a single timepoint at the end of the study (endpoint groups). For the weekly LDF groups, starting in Week 2, images of the incision site were taken during the LDF procedure prior to making an incision to assess the wound from the previous week. Blood samples were collected from the submandibular vein of all mice under anesthesia (at the end of the LDF procedure for the weekly groups). Blood samples were centrifuged at 2000 ×*g* for 10 min, and the isolated serum was stored at −80°C until analysis with an enzyme-linked immunosorbent assay (ELISA). At five days after each of the first three LDF procedures, gait patterns were assessed using high-speed video. Immediately following the last LDF procedure and serum collection, mice were euthanized using CO_2_ asphyxiation followed by cervical dislocation.

### 2.2 Tibial perfusion

**Fig. 2:**
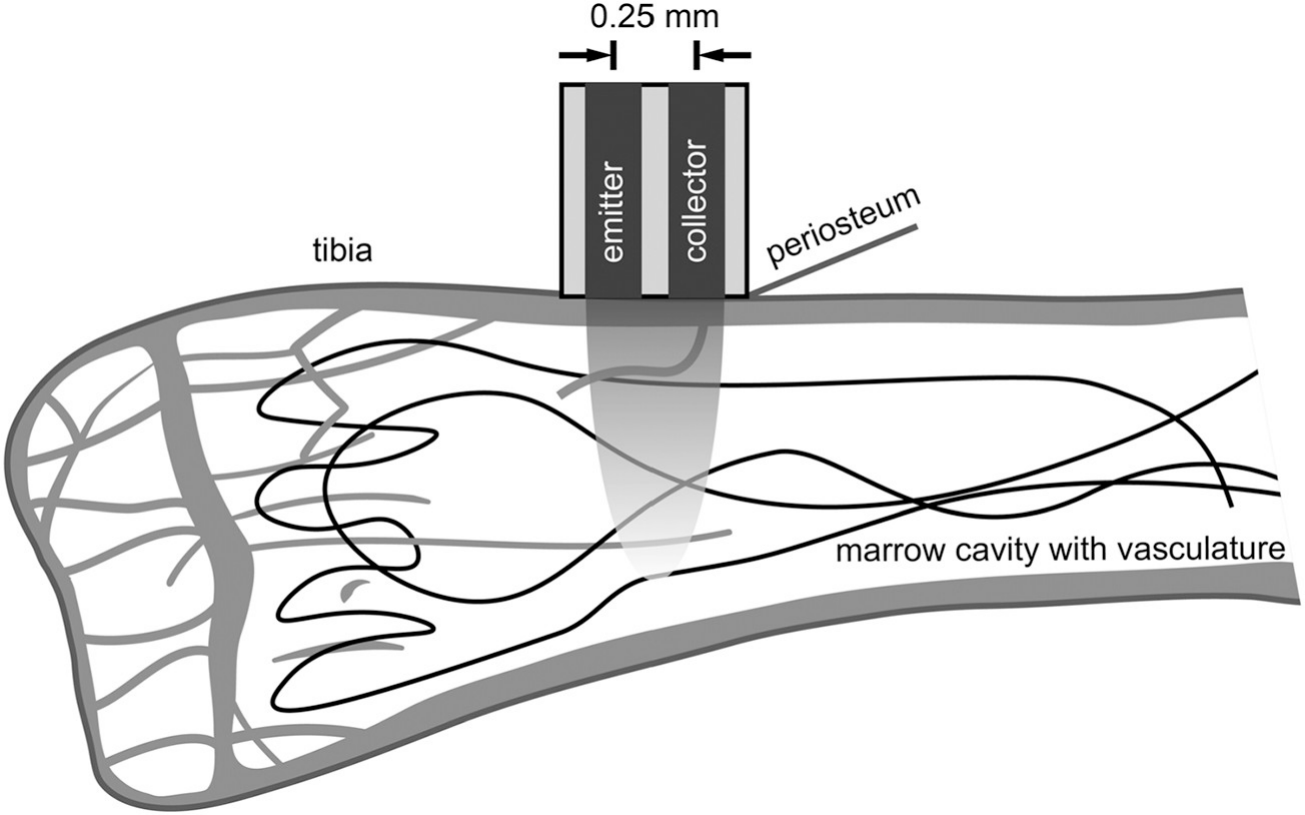
Schematic of LDF setup for bone perfusion measurements in the proximal tibia. The probe, placed on the tibial surface, emitted 785-nm light that scattered through a region of underlying tissue (represented by parabolic shading) and experienced Doppler shifts, some of which was scattered back to the collection probe, where it was measured.

All mice were fasted for 6–8 h before each LDF procedure. Anesthesia was induced and maintained with isoflurane (2%) in pure oxygen throughout the procedure (about 15 min). After anesthesia induction, the fur over the right knee was shaved, mice were placed supine on a heated pad, and the right leg was taped to the surgical platform. A 2–5-mm long incision was made over the anteromedial surface of the right proximal tibial metaphysis, the bone was exposed, and a small region of the periosteum was scraped away. LDF measurements were recorded using an LDF monitor with a 785-nm light source (MoorVMS-LDF, Moor Instruments Ltd., Axminster, UK) and a 3-kHz lowpass filter. A VP4 Needle Probe (0.8 mm outer diameter, 0.25 mm fiber separation) was placed directly on the exposed bone surface (Fig. 2) and held in place using a micromanipulator (MM3-ALL, World Precision Instruments, Sarasota, FL) to reduce signal noise from probe movement. Each weekly measurement was composed of the weighted mean of three 30-s readings, with repositioning of the probe between readings. Readings that contained noisy data with spikes or deviated substantially from a steady value (i.e., had a nonzero slope) were re-taken. The incisions were closed using VetBond™ tissue glue (3M, St. Paul, MN) and covered with triple antibiotic cream.

### 2.3 Femoral ligation validation

At the end of the study, just prior to euthanasia, additional LDF measurements were performed in a subset of mice (n = 12) during arterial ligation to confirm the association of LDF perfusion measurements with changes in blood supply to the bone. While still anesthetized during the final LDF procedure, the skin incision over the proximal tibia was extended to expose the entire inner thigh and the femoral artery. The LDF probe was again positioned over the tibial metaphysis, and a suture was tied around the femoral artery but not tightened. A 30-s baseline measurement was taken, the suture was tightened to ligate the artery, and another 30-s measurement was recorded. The reduction in tibial perfusion was calculated as the ratio of the ligated measurement to the baseline measurement, expressed as a percent.

### 2.4 Wound area

As mentioned above, for the weekly LDF groups, pictures were taken of the incision wounds immediately prior to each LDF procedure to assess localized inflammation and healing at the wound site from the procedure performed in the preceding week. Wound area was calculated by tracing the edge of the wound in ImageJ (version 1.51k, National Institutes of Health, Bethesda, MD). The wound was considered closed if no moist granulation tissue was visible and the wound was covered with new epithelium (Goova et al., 2001).

### 2.5 Serum concentration of interleukin 6

Systemic inflammation was examined by quantifying serum concentrations of the proinflammatory marker interleukin 6 (IL-6) with an ELISA (IL-6 Mouse ELISA kit, KMC0061, Invitrogen, Carlsbad, CA). A subset of the serum samples (Table 1) was prepared according to the manufacturer's instructions and measured using a plate reader (Synergy H1, BioTek Instruments, Inc., Winooski, VT). Due to limited serum, some samples were diluted 2–4 times to allow samples to be run in duplicate. Since no increase in IL-6 was detected in this subset, which included serum from all groups at each timepoint, the remaining serum samples were not analyzed.

**Table 1:** Serum concentrations of interleukin-6 in a subset of mice.

Group	Week 1	Week 2	Week 3	Week 4
Weekly-exercise	ND, n = 1	ND, n = 3	ND, n = 3	ND, n = 3
Weekly-sedentary	ND, n = 154.0 pg/mL, n = 1	ND, n = 1	ND, n = 3	ND, n = 3
Endpoint-exercise	ND, n = 2	ND, n = 2	ND, n = 2	ND, n = 2
Endpoint-sedentary	ND, n = 3	ND, n = 2	ND, n = 2	ND, n = 252.1 pg/mL, n = 1

ND: not detected.

### 2.6 Gait pattern analysis

**Fig. 3:**
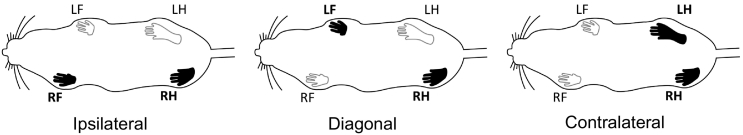
Schematic of the ipsilateral (RF-RH), diagonal (LF-RH), and contralateral (LH-RH) phase dispersions used for gait analysis. Relevant limbs relative to the LDF limb (RH) shaded in black. L = left, R = right, F = forelimb, H = hindlimb.

The effect of the LDF procedures on interlimb coordination was examined weekly in all mice, five days after each procedure day, because limping or other gait asymmetries could alter the strain experienced by hindlimb bones and confound bone outcome measures (Frost, 2003). During a short treadmill session (12 m/min for 60 s), high-speed video was collected in the sagittal plane at 240 frames per second (HERO4, GoPro, Inc., San Mateo, CA). Gait was analyzed using Kinovea (version 0.8, Kinovea Open Source Project) to quantify duty cycle for both hindlimbs and phase dispersions for ipsilateral, diagonal, and contralateral limbs with relation to the LDF limb (right hindlimb) (Fig. 3) (Leblond et al., 2003; Kloos et al., 2005; Redondo-Castro et al., 2013). Duty cycle for a given limb is the ratio of the time that limb is on the ground (measured from paw strike to lift off) to the total time of an entire gait cycle (measured from paw strike to paw strike). Phase dispersion between two limbs is a measure of the time between paw strikes for those two limbs within a gait cycle. Five consecutive gait cycles were analyzed for each treadmill session. Duty cycle and phase dispersion were averaged over the five gait cycles at each timepoint.

### 2.7 Statistical analysis

All data analyses were performed using SAS (SAS University Edition v. 9.4, SAS Institute Inc., Cary, NC) with a significance level of 0.05. Models were chosen to answer five questions: 1) Does performing weekly LDF procedures affect bone perfusion? To answer this question, LDF data from the final timepoint (Week 4) were compared across LDF frequency (weekly, endpoint) and exercise regimen (sedentary, exercise) using a two-way ANOVA with interaction. Tukey's post-hoc tests were used to compare group means. 2) Does exercise affect bone perfusion? For this question, LDF data for the weekly group were compared across exercise regimen and timepoint (Weeks 1–4) to examine differences between exercise groups within each timepoint (e.g., sedentary vs. exercise at Week 1). A mixed effects general linear model (procedure MIXED) with interaction was used, with exercise group as a fixed factor and timepoint as a repeated factor. The covariance matrix was modeled using compound symmetry. Exercise effect differences were calculated based on least squares means (LSM) with Tukey-Kramer adjustments for multiple comparisons. For the endpoint-only group (Week 4 data), the effect of exercise was examined with one-way ANOVA. 3) Do weekly LDF procedures alter exercise effects on bone perfusion? Effect differences between timepoints (e.g., Week 1 vs. Week 2) were evaluated in the same mixed effects model using LSM with Tukey-Kramer adjustments for multiple comparisons. 4) Do LDF readings directly correspond to changes in blood supply, assessed by femoral artery ligation? LDF data before and after the femoral artery was ligated were compared using a paired *t*-test. 5) Do weekly LDF procedures alter gait and interlimb coordination? This question was addressed by comparing gait parameters between LDF groups within exercise groups and timepoints (e.g., weekly sedentary vs. endpoint sedentary at Week 1). Four gait parameters were compared across LDF groups, exercise groups, and timepoints (Weeks 2–4) with mixed effects linear models (procedure MIXED) with interaction, where LDF frequency and exercise regimen were fixed factors and timepoint was a repeated measure. The covariance matrices were modeled using either compound symmetry (duty cycle, contralateral phase dispersion) or a first-order autoregressive (diagonal and ipsilateral phase dispersion), based on which covariance matrix yielded a lower corrected Akaike's Information Criterion. Effect differences were calculated based on LSM with Tukey-Kramer adjustments for multiple comparisons. All data are presented as mean ± standard deviation, except LDF and gait data, which are presented as LSM ± 95% confidence interval.

## 3 Results

### 3.1 Tibial perfusion

**Fig. 4:**
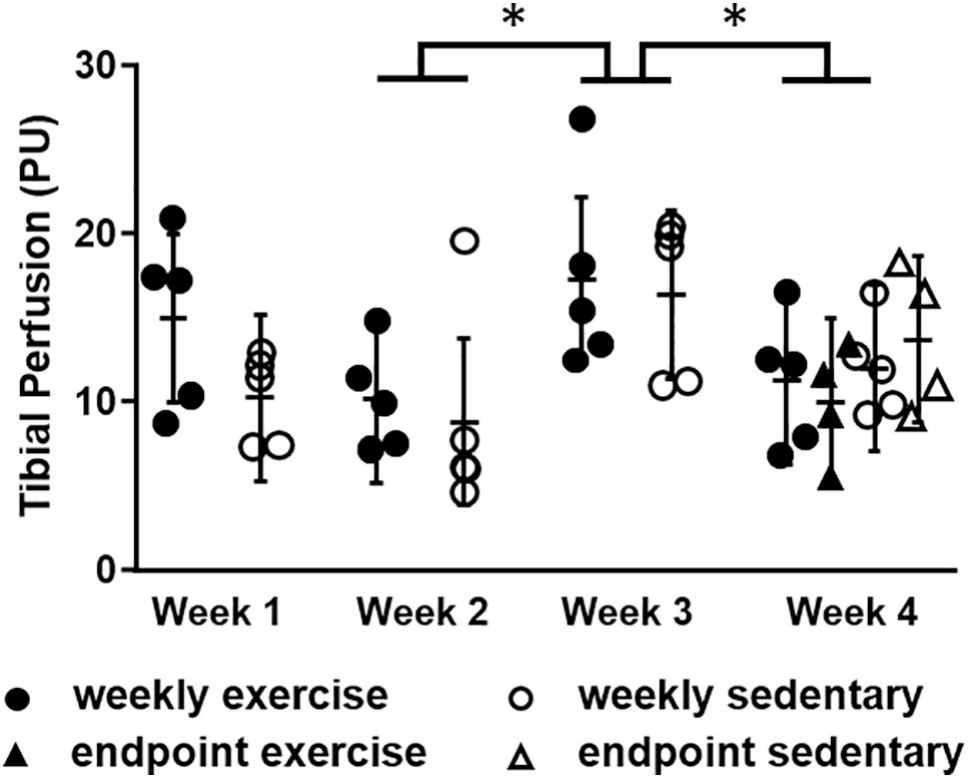
At Week 4, tibial perfusion was similar between weekly and endpoint-only groups. Sedentary and exercise groups had similar perfusion at every timepoint. LDF was sensitive to transient perfusion changes, measuring higher in the weekly groups in Week 3 compared to Week 2 and Week 4. PU = perfusion units. Data presented as least squares mean ± 95% confidence interval. *p < 0.05 Week 3 vs. Weeks 2 and 4.

At Week 4, bone perfusion was similar between the weekly and endpoint-only groups (p = 0.92), showing that our modified LDF procedure can be performed weekly without affecting perfusion, the primary outcome of interest (Fig. 4). With weekly LDF procedures, treadmill exercise did not affect perfusion measurements, compared to the sedentary group, at any timepoint (p = 0.11 Week 1, p = 0.64 Week 2, p = 0.76 Week 3, and p = 0.78 Week 4). Similarly, with endpoint-only LDF procedures, perfusion also did not differ significantly between exercise and sedentary groups at Week 4 (p = 0.49), suggesting weekly LDF procedures did not mask an exercise effect on perfusion. Perfusion was higher in weekly-sedentary and weekly-exercise groups during Week 3 compared to weekly-sedentary and weekly-exercise groups during Weeks 2 and 4, indicating that the technique is sensitive to transient perfusion changes regardless of exercise.

The average standard deviation over individual 30-s long recordings was 2.3 PU (18% of the mean of each recording). These variations may result from physiologic changes in blood supply within the tibia, small movements of the probe during the 30-s recording (e.g., due to animal breathing, etc.), and/or equipment noise. The average standard deviation over the three recordings' means was 3.1 PU (24% of the mean), with variations resulting primarily from slight differences in probe placement between the recordings.

### 3.2 Femoral ligation validation

**Fig. 5:**
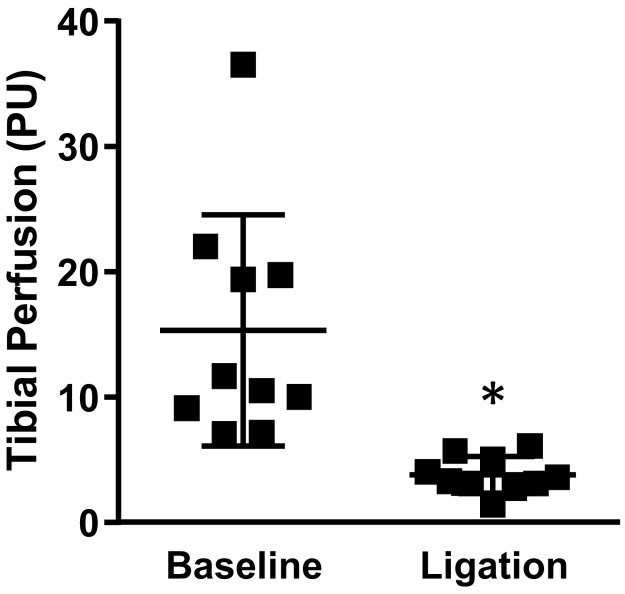
After ligation of the femoral artery, tibial perfusion dropped to 31 ± 19% of the baseline value. PU = perfusion units. *p < 0.05 ligation vs. baseline.

After the femoral artery was ligated, perfusion in the tibia dropped rapidly from 15.3 ± 9.2 perfusion units (PU) to 3.8 ± 1.4 PU within 30 s (31 ± 19% of the baseline perfusion, p = 0.004) (Fig. 5), validating that the LDF perfusion measurements are directly associated with blood supply within the bone. Two measurements were not included in the analysis, because the LDF probe slipped off the tibia when the ligature was tightened.

### 3.3 Wound area

**Fig. 6:**
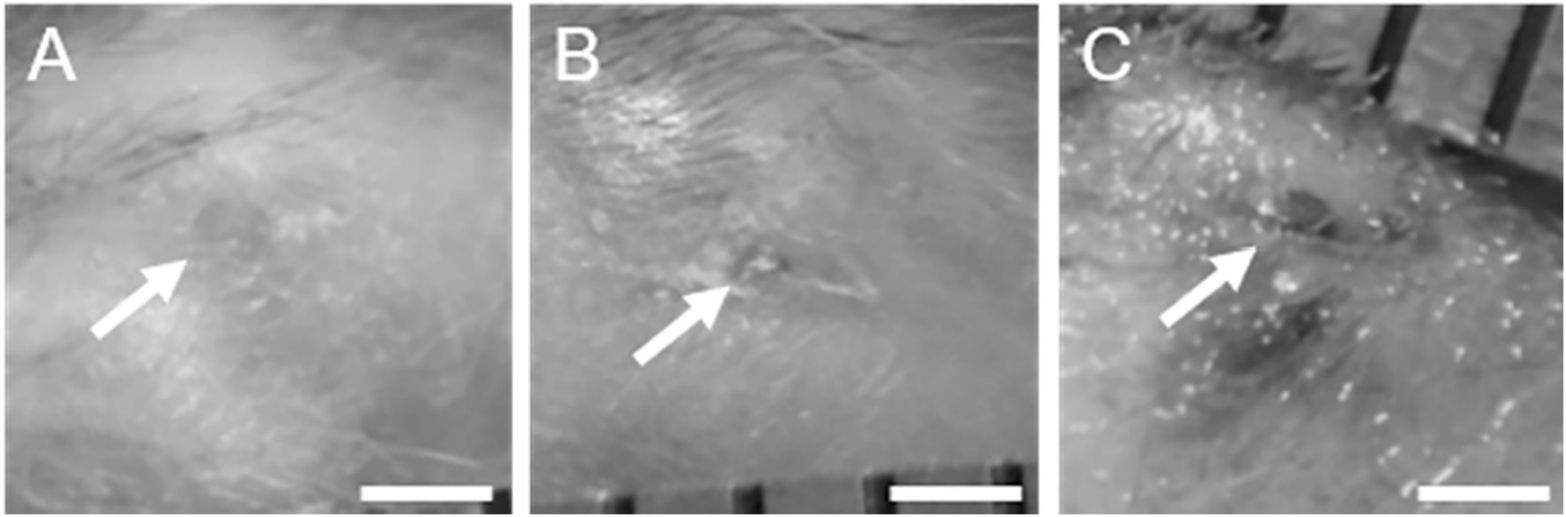
Example images of the incision wound site (arrows) from the weekly LDF group taken one week following the procedure. Incisions were either A) fully closed or B) closed with small, dry granulation tissue. C) Only one incision did not fully heal, but it was healed by the following week. Scale bars are 1 mm.

Wound images taken one week after each weekly LDF procedure showed minimal signs of inflammation for all but one incision for both exercise and sedentary groups, with either full closure (Fig. 6A) or a small, dry scab (Fig. 6B) resulting in zero wound area recorded. Wet granulation tissue was observed in only one incision site, for a sedentary mouse in Week 2 (Fig. 6C, wound area = 0.39 mm^2^), and the wound was healed within the subsequent week.

### 3.4 Serum concentration of IL-6

Circulating levels of proinflammatory marker IL-6 were below the detectable limit for all tested animals at each timepoint, except one mouse in Week 1 and one mouse in Week 4 (Table 1). Since the lower threshold of the test is 7.8 pg/mL, and serum samples were diluted up to four times to allow for duplicate measurements, serum levels of IL-6 were below 31.2 pg/mL. These levels agree with normal physiologic concentrations of IL-6, which are below 100 pg/mL in C57Bl/6J mice (Amar et al., 2007; Krause et al., 2012), suggesting the mice in our study experienced little to no systemic inflammation in response to the weekly LDF procedures. Pathologic inflammation can increase IL-6 levels up to 200–1000 pg/mL (Amar et al., 2007; Krause et al., 2012).

### 3.5 Gait pattern analysis

**Fig. 7:**
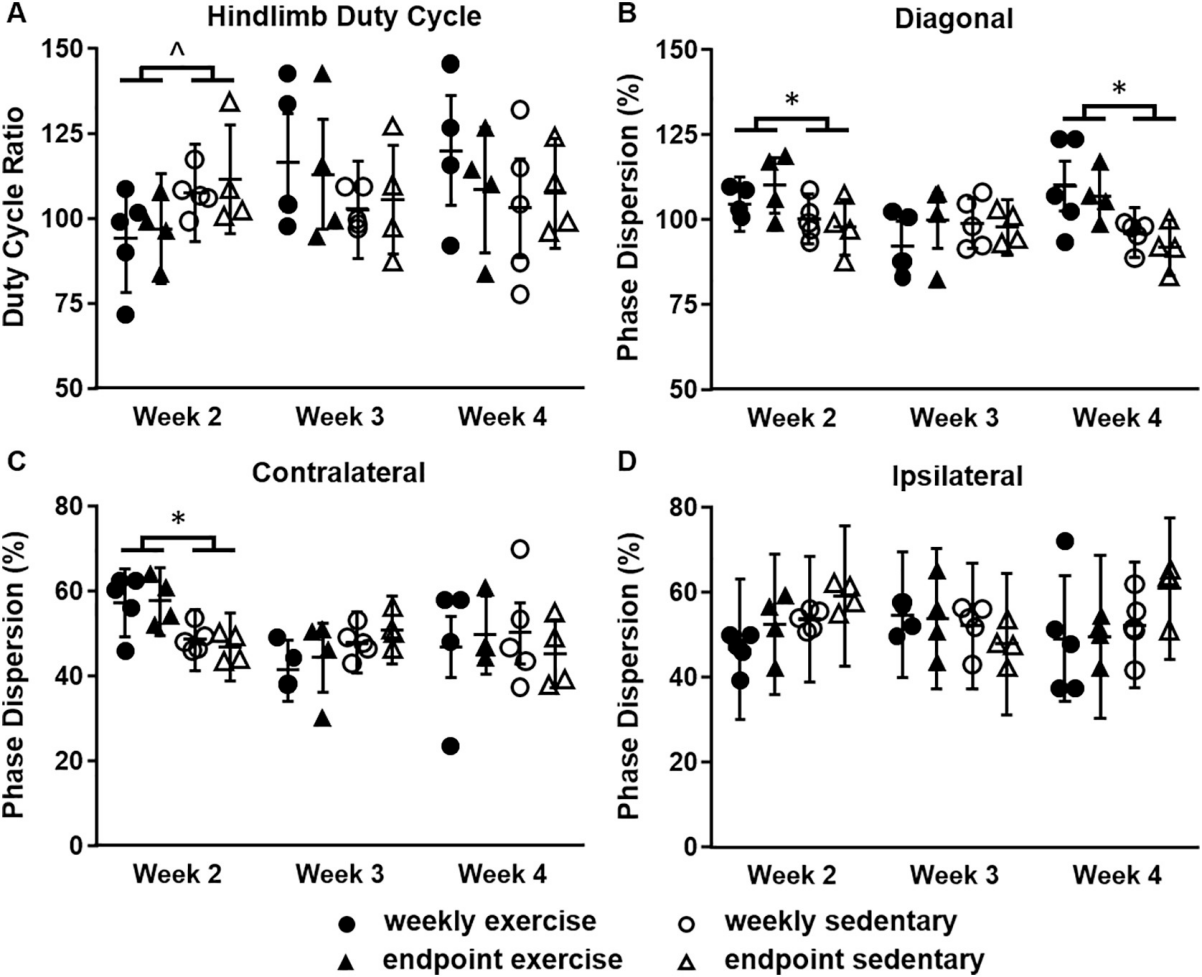
Treadmill locomotion patterns involving the LDF affected limb (right hindlimb). Gait patterns were not significantly different between weekly and endpoint-only groups at any timepoint for A) hindlimb duty cycle ratio, B) diagonal phase dispersion, C) contralateral phase dispersion, or D) ipsilateral phase dispersion. Diagonal and contralateral phase dispersion were lower in exercise than sedentary groups at some timepoints. Data are presented as least squares mean ± 95% confidence interval. *p < 0.05 exercise vs. sedentary. ^p = 0.066 exercise vs. sedentary.

Weekly LDF procedures did not affect gait parameters during treadmill locomotion. Limb coordination did not differ between weekly and endpoint-only groups at any timepoint for either sedentary or exercise groups (Fig. 7). Sedentary groups did have small alterations in gait parameters in Week 2 compared to exercise groups (hindlimb duty cycle and diagonal and contralateral phase dispersion), possibly indicating slight discomfort or unfamiliarity with treadmill locomotion. Diagonal phase dispersion was lower in the exercise groups compared to sedentary groups during Week 4 (8.6% lower, p = 0.012).

## 4 Discussion

Our minimally invasive laser Doppler flowmetry technique measured in vivo intraosseous perfusion in the tibia weekly without inducing localized or systemic inflammation. LDF measures of tibial perfusion were similar between groups that received weekly procedures and groups that received only an endpoint procedure, indicating that the procedure itself did not impact measurements of intraosseous perfusion. A previous study demonstrated that LDF could be used to quantify blood perfusion in murine tibiae but noted that signs of inflammation were observed at the incision site up to three months following the procedure and suggested that it be used as an endpoint-only measure to avoid influencing bone outcomes (Roche et al., 2013). In this study, we limited the invasiveness of the procedure by reducing the incision size and preserving muscle tissue, thereby enabling repeated measurements to be performed without causing chronic increases in proinflammatory marker IL-6. Furthermore, all but one incision from the procedures were fully closed within the one-week period before the next procedure, and no visual signs of inflammation were observed throughout the study. The fast healing times observed in the small incisions from this procedure (2–5 mm) are consistent with wound closure studies, where even large (9–28 mm^2^), unclosed dermal biopsies heal in 7–14 days (Keylock et al., 2008).

The femoral ligation test confirmed that LDF measures are sensitive to changes in blood supply and thus are directly related to perfusion within the underlying cortical bone and marrow space. We demonstrated that femoral artery ligation reliably decreased the measured perfusion to about 3.8 PU (31% of baseline) within 30 s in all mice. The variation in perfusion observed in each 30-s long measurement was due primarily to probe movement (when the mouse breathes), equipment noise, and changes in blood supply within the tibia. Measurement duration was not optimized in this study, but the variation around the mean value appears constant for most readings. The 30-s measurement is fairly short yet provides enough data to determine if the reading deviates from a steady value (i.e., has a substantial slope) or is excessively noisy (i.e., with large spikes), both of which are criteria for excluding the reading and repositioning the probe. Inter-measurement variation can be minimized with better probe placement as the researcher becomes more familiar with the LDF procedure. In a subsequent study, we used only two measurements per bone and achieved a standard deviation of 2.1 PU, only 15% of the mean value (Hanne et al., 2019b).

Cortical thickness in the tibia affects the depth into the marrow space that LDF can measure. Another study quantified this relationship in the murine tibia and reported that small variations in cortical thickness had minimal effect on LDF perfusion measurements (Roche et al., 2013), suggesting that LDF can be used to track longitudinal changes in bone perfusion. Because cortical thickness in the tibial diaphysis changes by about 10% from skeletal maturity at 16 weeks of age to 52 weeks of age (Ferguson et al., 2003), and can differ by sex (Somerville et al., 2004), longitudinal LDF measurements should only be performed in age- and sex-matched subjects. Finally, we found no interaction effect of daily treadmill exercise on LDF readings. Taken together, these results suggest that our minimally invasive LDF procedure is suitable for monitoring and comparing blood perfusion longitudinally in murine studies involving exercise therapy. This procedure can be used to track changes to osteovascular function, which is known to play an important role in bone development, remodeling, and repair (Marks and Odgren, 2002; Gerber et al., 1999), yet remains under-studied.

In addition to providing aerobic exercise, treadmill activity also mechanically loads the bones and increases the functional strain experienced by the bones (Wallace et al., 2007; Prasad et al., 2010; Berman et al., 2019). Even slight changes to functional strain can affect osteogenesis (Frost, 2003; Ellman et al., 2013) and angiogenesis (Yao et al., 2004; Steward et al., 2016). Since other studies have shown that changes to gait kinematics and limb patterning affect bone strain (Prasad et al., 2010; Hurwitz et al., 1998), we were concerned the LDF procedure could affect locomotion patterns and confound exercise effects by altering functional strain. We found no differences in duty cycle or interlimb coordination between weekly and endpoint-only groups at any timepoint, indicating that weekly LDF measurements do not alter gait patterns (and thus functional strain) during treadmill exercise.

Although not the main focus of this study, aerobic treadmill exercise was anticipated to cause increased tibial perfusion over time due to vascular growth and adaptation, as we have previously found in studies using the same exercise regimen and LDF technique (Hanne et al., 2019a; Hanne et al., 2019b). Perfusion is a functional measure of not only the amount and direction of blood flow but also vascular permeability and capillary density (Griffith, 2014). Treadmill exercise may be affecting the size, number, or cellular makeup of the vasculature without causing changes in perfusion. Rats that performed a similar treadmill routine for two weeks had a 19% increase in the number but not total area of blood vessels in the proximal tibial metaphysis compared to the sedentary group (Yao et al., 2004). A more rigorous aerobic exercise intervention, such as free access to running wheels where mice will run 4–10 km daily (Gertz et al., 2006; Styner et al., 2017), may have a larger and more detectible effect on perfusion. Nevertheless, our study confirmed that weekly LDF procedures will not confound perfusion measurements in future exercise studies.

Stress, like inflammation and aerobic exercise, can also affect vascular function. Neuropeptide Y, which is expressed during stress response, has been shown to be both angiogenic and vasoconstrictive, which could increase blood pressure and the amount of vasculature in bone (Kuo and Zukowska, 2007). Although we found almost no detectible increases in IL-6 or visible signs of inflammation at the incision site, both sedentary and exercise groups had a significant increase in tibial perfusion in the third week that was resolved by the fourth week. Stress may have played a role in increasing perfusion in the third week; all mice were handled daily for either treadmill exercise or the sedentary treadmill activity and had blood drawn weekly, which could induce a stress response. The increased perfusion lasted only one week and was present in both exercise and sedentary groups. The low serum yield in these small animals limited our assessments to one inflammation marker in a subset of mice – other markers of inflammation may have been elevated, or IL-6 may have been elevated in the subset of mice that were not measured. IL-6 is known to be elevated following musculoskeletal trauma (Reikeras et al., 2014) and has been shown to stimulate bone resorption (Ishimi et al., 1990), which would preclude the use of LDF as a weekly tool in most bone experiments.

This study had several limitations that warrant attention in future studies. A primary concern of this technique is the removal of a small area of the periosteum, a highly vascularized tissue that contains osteoblast precursor cells (Colnot et al., 2012). LDF measures of intracortical perfusion in the tibiae of juvenile ewes dropped by 25% immediately following the removal of the periosteum from the medial aspect (Kowalski et al., 1996). Although the amount of periosteum removed in our procedure is small (about the size of our probe, 0.5 mm^2^ area), the effects of periosteal removal were not examined and may affect bone tissue function. The effects of weekly LDF procedures on bone remodeling and homeostasis were not examined in this study. This study did not compare LDF results to other promising emerging techniques for examining osteovasculature in vivo. Several new higher resolution PET scans can be used in rodent bones (Tomlinson et al., 2012), and emerging MRI techniques (e.g., blood oxygen level-dependent MRI and intravoxel incoherent motion MRI (Griffith, 2014)) greatly improve resolution and do not require contrast agents, but these techniques remain prohibitively expensive.

## 5 Conclusions

Weekly LDF procedures performed over four weeks did not induce measurable signs of inflammation or significantly alter gait patterns during treadmill exercise. Unlike other existing methods used for measuring the vascular network in bone, this procedure can be performed in vivo, is repeatable without confounding study controls, is relatively simple to perform, and is inexpensive. Monitoring intraosseous perfusion serially with LDF provides a functional measure of blood flow, enabling researchers to track changes to the osteovasculature noninvasively during disease progression and interventions.

## CRediT authorship contribution statement

**Nicholas J. Hanne:**Conceptualization, Methodology, Validation, Formal analysis, Writing - original draft, Writing - review & editing, Visualization.**Elizabeth D. Easter:**Conceptualization, Methodology, Validation, Investigation, Writing - original draft, Funding acquisition.**Jacqueline H. Cole:**Conceptualization, Formal analysis, Writing - review & editing, Supervision, Project administration, Funding acquisition.

## References

[bb0005] Amar S., Zhou Q., Shaik-Dasthagirisaheb Y., Leeman S. (2007). Diet-induced obesity in mice causes changes in immune responses and bone loss manifested by bacterial challenge. Proc. Natl. Acad. Sci. U. S. A..

[bb0010] Au J.T., Craig G., Longo V., Zanzonico P., Mason M., Fong Y., Allen P.J. (2013). Gold nanoparticles provide bright long-lasting vascular contrast for CT imaging. Am. J. Roentgenol..

[bb0015] Barou O., Mekraldi S., Vico L., Boivin G., Alexandre C., Lafage-Proust M.H. (2002). Relationships between trabecular bone remodeling and bone vascularization: a quantitative study. Bone.

[bb0020] Berman A.G., Hinton M.J., Wallace J.M. (2019). Treadmill running and targeted tibial loading differentially improve bone mass in mice. Bone Rep.

[bb0025] Boerckel J.D., Uhrig B.A., Willett N.J., Huebsch N., Guldberg R.E. (2011). Mechanical regulation of vascular growth and tissue regeneration in vivo. Proc. Natl. Acad. Sci. U. S. A..

[bb0030] Briers D., Duncan D.D., Hirst E.R., Kirkpatrick S.J., Larsson M., Steenbergen W., Stromberg T., Thompson O.B. (2013). Laser speckle contrast imaging: theoretical and practical limitations. J. Biomed. Opt..

[bb0035] Clark D.P., Ghaghada K., Moding E.J., Kirsch D.G., Badea C.T. (2013). In vivo characterization of tumor vasculature using iodine and gold nanoparticles and dual energy micro-CT. Phys. Med. Biol..

[bb0040] Colnot C., Zhang X., Tate M.L.K. (2012). Current insights on the regenerative potential of the periosteum: molecular, cellular, and endogenous engineering approaches. J. Orthop. Res..

[bb0045] Dyke J.P., Aaron R.K. (2009). Noninvasive methods of measuring bone blood perfusion. Ann. N. Y. Acad. Sci..

[bb0050] Ellman R., Spatz J., Cloutier A., Palme R., Christiansen B.A., Bouxsein M.L. (2013). Partial reductions in mechanical loading yield proportional changes in bone density, bone architecture, and muscle mass. J. Bone Miner. Res..

[bb0055] Farhat G.N., Newman A.B., Sutton-Tyrrell K., Matthews K.A., Boudreau R., Schwartz A.V., Harris T., Tylavsky F., Visser M., Cauley J.A., for the H.A. Study (2007). The association of bone mineral density measures with incident cardiovascular disease in older adults. Osteoporos. Int..

[bb0060] Ferguson V.L., Ayers R.A., Bateman T.A., Simske S.J. (2003). Bone development and age-related bone loss in male C57BL/6J mice. Bone.

[bb0065] Frost H.M. (2003). Bone’s Mechanostat: a 2003 update. Anat. Rec..

[bb0070] Gerber H.-P., Vu T.H., Ryan A.M., Kowalski J., Werb Z., Ferrara N. (1999). VEGF couples hypertrophic cartilage remodeling, ossification and angiogenesis during endochondral bone formation. Nat. Med..

[bb0075] Gertz K., Priller J., Kronenberg G., Fink K.B., Winter B., Schröck H., Ji S., Milosevic M., Harms C., Böhm M., Dirnagl U., Laufs U., Endres M. (2006). Physical activity improves long-term stroke outcome via endothelial nitric oxide synthase–dependent augmentation of neovascularization and cerebral blood flow. Circ. Res..

[bb0080] Goova M.T., Li J., Kislinger T., Qu W. (2001). Blockade of receptor for advanced blycation end-products restores effective wound healing in diabetic mice. Am. J. Pathol..

[bb0085] Griffith J.F. (2014). Imaging vasculature of bone. Journal of Orthopaedic Translation.

[bb0090] Griffith J.F., Yeung D.K.W., Antonio G.E., Lee F.K.H., Hong A.W.L., Wong S.Y.S., Lau E.M.C., Leung P.C. (2005). Vertebral bone mineral density, marrow perfusion, and fat content in healthy men and men with osteoporosis: dynamic contrast-enhanced MR imaging and MR spectroscopy. Radiology.

[bb0095] Griffith J.F., Yeung D.K., Tsang P.H., Choi K.C., Kwok T.C., Ahuja A.T., Leung K.S., Leung P.C. (2008). Compromised bone marrow perfusion in osteoporosis. J. Bone Miner. Res..

[bb0100] Grundnes O., Reikerås O. (1992). Blood flow and mechanical properties of healing bone. Acta Orthop. Scand..

[bb0105] Hanne N.J., Steward A.J., Cox J.M., Easter E.D., Thornburg H.L., Sessions M.R., Pinnamaraju S.V., Cole J.H. (2019). High fat diet-induced obesity negatively affects whole bone bending strength but not cortical structure in the femur. BioRxiv.

[bb0110] Hanne N.J., Steward A.J., Geeroms C., Easter E.D., Thornburg H.L., Kerckhofs G., Parac-Vogt T., Sheng H., Cole J.H. (2019). Ischemic stroke reduces bone perfusion and alters osteovascular structure. BioRxiv.

[bb0115] Hellem S., Jacobsson L.S., Nilsson G.E., Lewis D.H. (1983). Measurement of microvascular blood flow in cancellous bone using laser Doppler flowmetry and 133Xe-clearance. Int. J. Oral Surg..

[bb0120] Hurwitz D.E., Sumner D.R., Andriacchi T.P., Sugar D.A. (1998). Dynamic knee loads during gait predict proximal tibial bone distribution. J. Biomech..

[bb0125] Ishimi Y., Miyaura C., Jin C.H., Akatsu T., Abe E., Nakamura Y., Yamaguchi A., Yoshiki S., Matsuda T., Hirano T. (1990). IL-6 is produced by osteoblasts and induces bone resorption. J. Immunol..

[bb0130] Kerckhofs G., Stegen S., van Gastel N., Sap A., Falgayrac G., Penel G., Durand M., Luyten F.P., Geris L., Vandamme K., Parac-Vogt T., Carmeliet G. (2018). Simultaneous three-dimensional visualization of mineralized and soft skeletal tissues by a novel microCT contrast agent with polyoxometalate structure. BIomater.

[bb0135] Keylock K.T., Vieira V.J., Wallig M.A., DiPietro L.A., Schrementi M., Woods J.A. (2008). Exercise accelerates cutaneous wound healing and decreases wound inflammation in aged mice. A. J. Physiol.-Reg. I..

[bb0140] Kloos A.D., Fisher L.C., Detloff M.R., Hassenzahl D.L., Basso D.M. (2005). Stepwise motor and all-or-none sensory recovery is associated with nonlinear sparing after incremental spinal cord injury in rats. Exp. Neurol..

[bb0145] Kowalski M.J., Schemitsch E.H., Kregor P.J., Senft D., Swiontkowski M.F. (1996). Effect of periosteal stripping on cortical bone perfusion: a laser doppler study in sheep. Calcif. Tissue Int..

[bb0150] Krause M. da S., Bittencourt A., de Bittencourt P.I.H., McClenaghan N.H., Flatt P.R., Murphy C., Newsholme P. (2012). Physiological concentrations of interleukin-6 directly promote insulin secretion, signal transduction, nitric oxide release, and redox status in a clonal pancreatic β-cell line and mouse islets. J. Endocrinol..

[bb0155] Kuo L.E., Zukowska Z. (2007). Stress, NPY and vascular remodeling: implications for stress-related diseases. Peptides.

[bb0160] Leblond H., L’Espérance M., Orsal D., Rossignol S. (2003). Treadmill locomotion in the intact and spinal mouse. J. Neurosci..

[bb0165] Marks S.C., Odgren P.R., Bilezikian J.P., Raisz L.G., Rodan G.A. (2002). Chapter 1 - structure and development of the skeleton. Principles of Bone Biology.

[bb0170] Melnyk M., Henke T., Claes L., Augat P. (2008). Revascularisation during fracture healing with soft tissue injury. Arch. Orthop. Trauma Surg..

[bb0175] Naves M., Rodríguez-García M., Díaz-López J.B., Gómez-Alonso C., Cannata-Andía J.B. (2008). Progression of vascular calcifications is associated with greater bone loss and increased bone fractures. Osteoporos. Int..

[bb0180] Nilsson G.E., Tenland T., Oberg P.A. (1980). Evaluation of a laser Doppler flowmeter for measurement of tissue blood flow. IEEE T. Biomed. Eng..

[bb0185] Okunieff P., Wang X., Rubin P., Finkelstein J.N., Constine L.S., Ding I. (1998). Radiation-induced changes in bone perfusion and angiogenesis. Int. J. Radiat. Oncol..

[bb0190] Parfitt A.M. (2000). The mechanism of coupling: a role for the vasculature. Bone.

[bb0195] Prasad J., Wiater B.P., Nork S.E., Bain S.D., Gross T.S. (2010). Characterizing gait induced normal strains in a murine tibia cortical bone defect model. J. Biomech..

[bb0200] Redondo-Castro E., Torres-Espín A., García-Alías G., Navarro X. (2013). Quantitative assessment of locomotion and interlimb coordination in rats after different spinal cord injuries. J. Neurosci. Methods.

[bb0205] Reeve J., Arlot M., Wootton R., Edouard C., Tellez M., Hesp R., Green J.R., Meunier P.J. (1988). Skeletal blood flow, iliac histomorphometry, and strontium kinetics in osteoporosis: a relationship between blood flow and corrected apposition rate. J. Clin. Endocrin. & Metab..

[bb0210] Reikeras O., Borgen P., Reseland J.E., Lyngstadaas S.P. (2014). Changes in serum cytokines in response to musculoskeletal surgical trauma. BMC Research Notes.

[bb0215] Roche B., David V., Vanden-Bossche A., Peyrin F., Malaval L., Vico L., Lafage-Proust M.-H. (2012). Structure and quantification of microvascularisation within mouse long bones: what and how should we measure?. Bone.

[bb0220] Roche B., Vanden-Bossche A., Normand M., Malaval L., Vico L., Lafage-Proust M.-H. (2013). Validated laser Doppler protocol for measurement of mouse bone blood perfusion — response to age or ovariectomy differs with genetic background. Bone.

[bb0225] Serrat M.A. (2009). Measuring bone blood supply in mice using fluorescent microspheres. Nat. Protoc..

[bb0230] Somerville J.M., Aspden R.M., Armour K.E., Armour K.J., Reid D.M. (2004). Growth of C57BL/6 mice and the material and mechanical properties of cortical bone from the tibia. Calcif. Tissue Int..

[bb0235] Steward A.J., Cole J.H., Ligler F.S., Loboa E. (2016). Mechanical and vascular cues synergistically enhance osteogenesis in human mesenchymal stem cells. Tissue Eng. Part A..

[bb0240] Styner M., Pagnotti G.M., McGrath C., Wu X., Sen B., Uzer G., Xie Z., Zong X., Styner M.A., Rubin C.T., Rubin J. (2017). Exercise decreases marrow adipose tissue through ß-oxidation in obese running mice. J. Bone Miner. Res..

[bb0245] Swiontkowski M.F. (1991). Laser Doppler flowmetry—development and clinical application. Iowa Orthop. J..

[bb0250] Tomlinson R.E., Silva M.J., Shoghi K.I. (2012). Quantification of skeletal blood flow and fluoride metabolism in rats using PET in a pre-clinical stress fracture model. Mol. Imaging Biol..

[bb0255] Tomlinson R.E., McKenzie J.A., Schmieder A.H., Wohl G.R., Lanza G.M., Silva M.J. (2013). Angiogenesis is required for stress fracture healing in rats. Bone.

[bb0260] Trueta J. (1963). The role of the vessels in osteogenesis. Bone Joint J..

[bb0265] Wallace A.L., Draper E.R.C., Strachan R.K., Mccarthy I.D., Hughes S.P.F. (1991). The effect of devascularisation upon early bone healing in dynamic external fixation. Bone Joint J.

[bb0270] Wallace J.M., Rajachar R.M., Allen M.R., Bloomfield S.A., Robey P.G., Young M.F., Kohn D.H. (2007). Exercise-induced changes in the cortical bone of growing mice are bone- and gender-specific. Bone.

[bb0275] Whiteside L.A., Lesker P.A., Simmons D.J. (1977). Measurement of regional bone and bone marrow blood flow in the rabbit using the hydrogen washout technique. Clin. Orthop. Relat. Res..

[bb0280] Yao Z., Lafage-Proust M.-H., Plouët J., Bloomfield S., Alexandre C., Vico L. (2004). Increase of both angiogenesis and bone mass in response to exercise depends on VEGF. J. Bone Miner. Res..

